# Selection of suitable hand gestures for reliable myoelectric human computer interface

**DOI:** 10.1186/s12938-015-0025-5

**Published:** 2015-04-09

**Authors:** Maria Claudia F Castro, Sridhar P Arjunan, Dinesh K Kumar

**Affiliations:** Electrical Engineering Department, Centro Universitário da FEI, Av. Humberto de A. C. Branco, 3.972, São Bernardo do Campo, SP 09850-901 Brazil; Biosignal Lab., School of Electrical and Computer Engineering, RMIT University, GPO Box 2476, Melbourne, VIC 3001 Australia

**Keywords:** Hand gesture, Finger flexion, Myoelectric signal, Frequency domain, Pattern recognition

## Abstract

**Background:**

Myoelectric controlled prosthetic hand requires machine based identification of hand gestures using surface electromyogram (sEMG) recorded from the forearm muscles. This study has observed that a sub-set of the hand gestures have to be selected for an accurate automated hand gesture recognition, and reports a method to select these gestures to maximize the sensitivity and specificity.

**Methods:**

Experiments were conducted where sEMG was recorded from the muscles of the forearm while subjects performed hand gestures and then was classified off-line. The performances of ten gestures were ranked using the proposed Positive–Negative Performance Measurement Index (PNM), generated by a series of confusion matrices.

**Results:**

When using all the ten gestures, the sensitivity and specificity was 80.0% and 97.8%. After ranking the gestures using the PNM, six gestures were selected and these gave sensitivity and specificity greater than 95% (96.5% and 99.3%); Hand open, Hand close, Little finger flexion, Ring finger flexion, Middle finger flexion and Thumb flexion.

**Conclusion:**

This work has shown that reliable myoelectric based human computer interface systems require careful selection of the gestures that have to be recognized and without such selection, the reliability is poor.

## Introduction

Powered prosthetic hands are used by trans-radial amputee and may also find applications for the elderly and frail people. Prosthetic hand control requires automatic recognition of the user commands for flexion/extension of the fingers and hand. Myoelectric based proportional control of the prosthetic hands provides the user with the natural and intuitive control of the device and is the desired option. These systems record surface electromyogram (sEMG) from the residual muscles of the forearm and classify the signals to identify the user command. It is important that such a system is reliable, non-invasive, and simple with small number of electrodes that can be used without requiring an expert to mount the system [1-3].

Many hand gestures require simultaneous contraction of multiple overlapping muscles and this makes it difficult to directly map the surface electromyogram (sEMG) to different hand and finger gestures. Thus, systems that have been reported in literature estimate the hand and finger commands by training the system for the user with a limited number of commands such as individual finger gestures [4-11] or some functional hand gestures [12-19]. Number of studies have analyzed the recordings to obtain the most suitable signal features and classification techniques. In general, the recordings are obtained from the forearm of the healthy subjects, and the technology so developed is translated for the amputee patients. After the signal classification technique is evaluated, the systems are customized for the individual users because of the large differences between people. However, in none of these studies, the selection of the various gestures have been examined and justified. Earlier works appear to select the gestures based on user requirements [28] but without any consideration to reducing error or improving the sensitivity and specificity. This limits the overall functionality of the prosthetic device and may also compromise on the overall reliability.

This work has investigated the relationship between the set of hand-gestures and the sensitivity and specificity of automated hand-gesture recognition, has observed that it is essential to carefully select these gestures for an accurate system and reports a method to select the most suitable gestures. From literature, there appear to be ten commonly used hand gestures for prosthetic hand and other similar applications; five individual finger gestures and five functional hand gestures. This study analyzed the sensitivity, specificity and accuracy for the recognition of these gestures, and developed an iterative technique to identify those set of gestures that give suitable sensitivity and specificity. A standard myoelectric classification system was developed based on commonly used signal recording, analysis and classification methods and used to conduct the experiments. Confusion matrix based index was developed to identify the suitable set of gestures by ranking these gestures based on the misclassification caused due to them.

The main outcome of this work is the demonstration of the method for selecting the suitable gestures for ensuring high sensitivity and selectivity, which are essential for controlling the prosthetic hand device. The significance of this study is the development of new technique to obtain the subset of most suitable hand-gestures from the larger set by ranking based on the confusion matrix. This would provide the suitable performance for classifying the hand-gestures. The paper uses existing signal processing, feature selection and classification methods of sEMG that have been reported in literature.

## Materials and methods

### Subjects

Experiments were conducted after receiving ethics approval from research ethics committee of São Judas Tadeu University (COEP - USJT - No.088/2011), and in accordance with the Helsinki accord (modified 2004). Four volunteers (average age 19), with no neurological or muscular disease or injury, participated in this study. They were given the plain language statement that explained the experiment and possible risk and the protocol was also explained to them orally. Verbal and written consent was obtained prior to conducting experiments.

### Equipment and recording procedures

**Figure 1:**
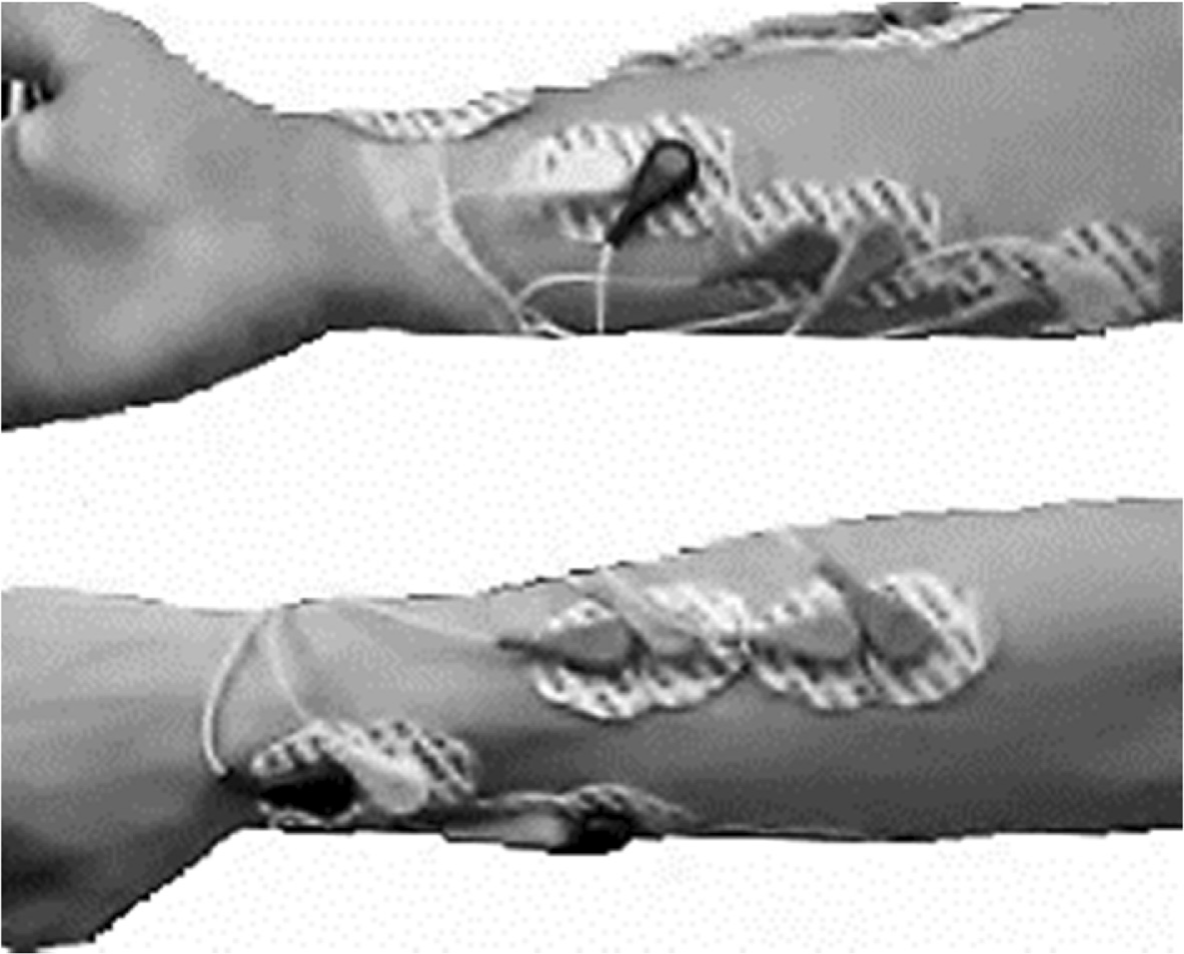
**Electrode positioning.**

Surface EMG signals were recorded using the PowerLab 16/30 (AdInstruments), with 1000 Hz sampling rate and 20–500 Hz filtering range, using eighth-order, switched-capacitance, Bessel type filter. Disposable adhesive electrodes from Medtrace in bipolar configuration with 20 mm inter-electrode distance were used. Five pairs of electrodes were placed based on the commonly accepted low density electrode placement for hand based myoelectric systems [20]. The preparation and placement of the electrodes was based on SENIAM recommendation [21] and were placed to record from forearm muscles: *flexor digitorum superficialis, palmaris longus, abductor pollicis longus, extensor digiti minimi and extensor communis digitorum*. A common ground electrode was placed on the radial styloid process (Figure 1).

### Experiment protocol

**Figure 2:**
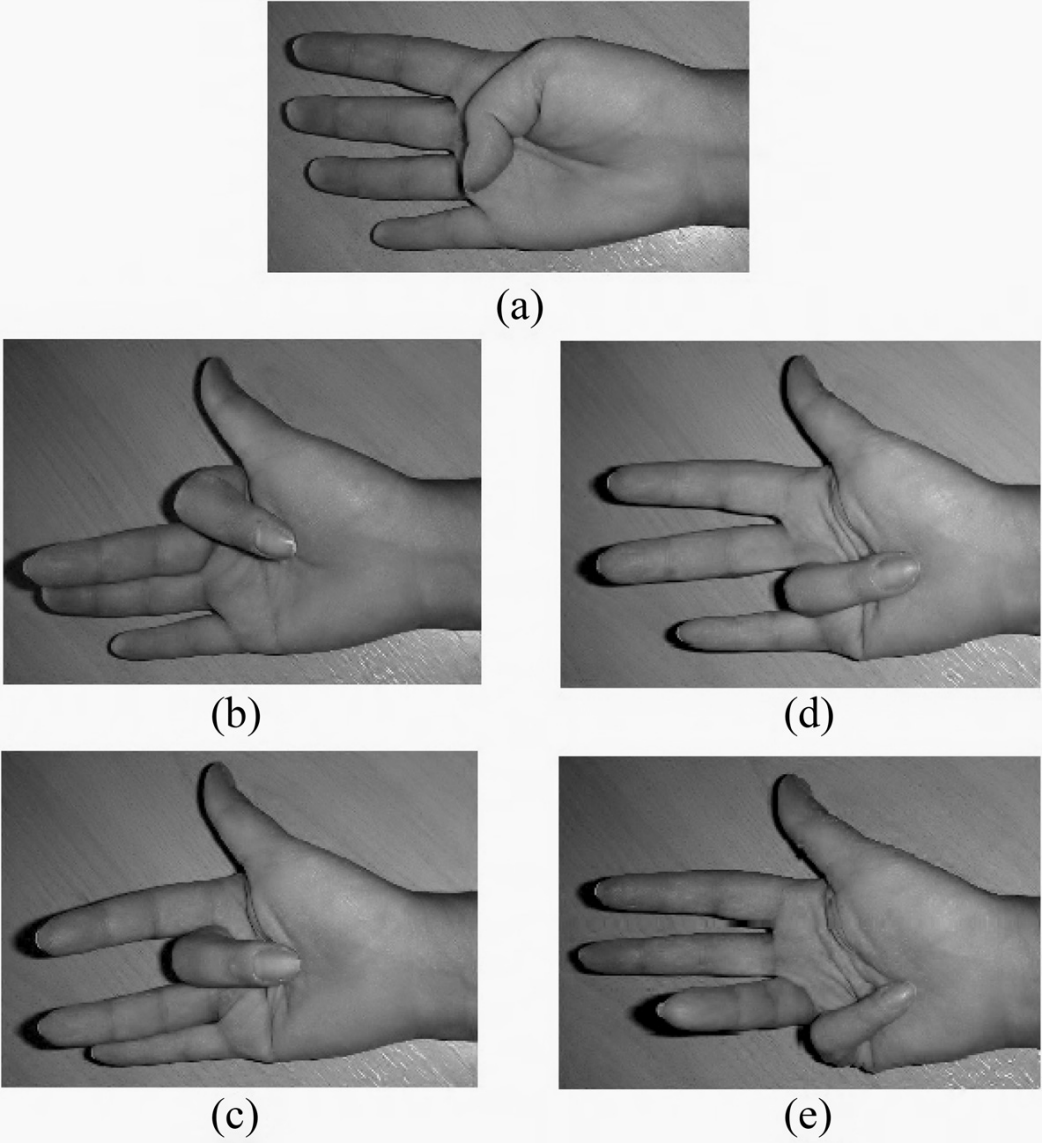
**Finger positioning: (a) Thumb, (b) Index finger, (c) Middle finger, (d) Ring finger, (e) Little finger.**

**Figure 3:**
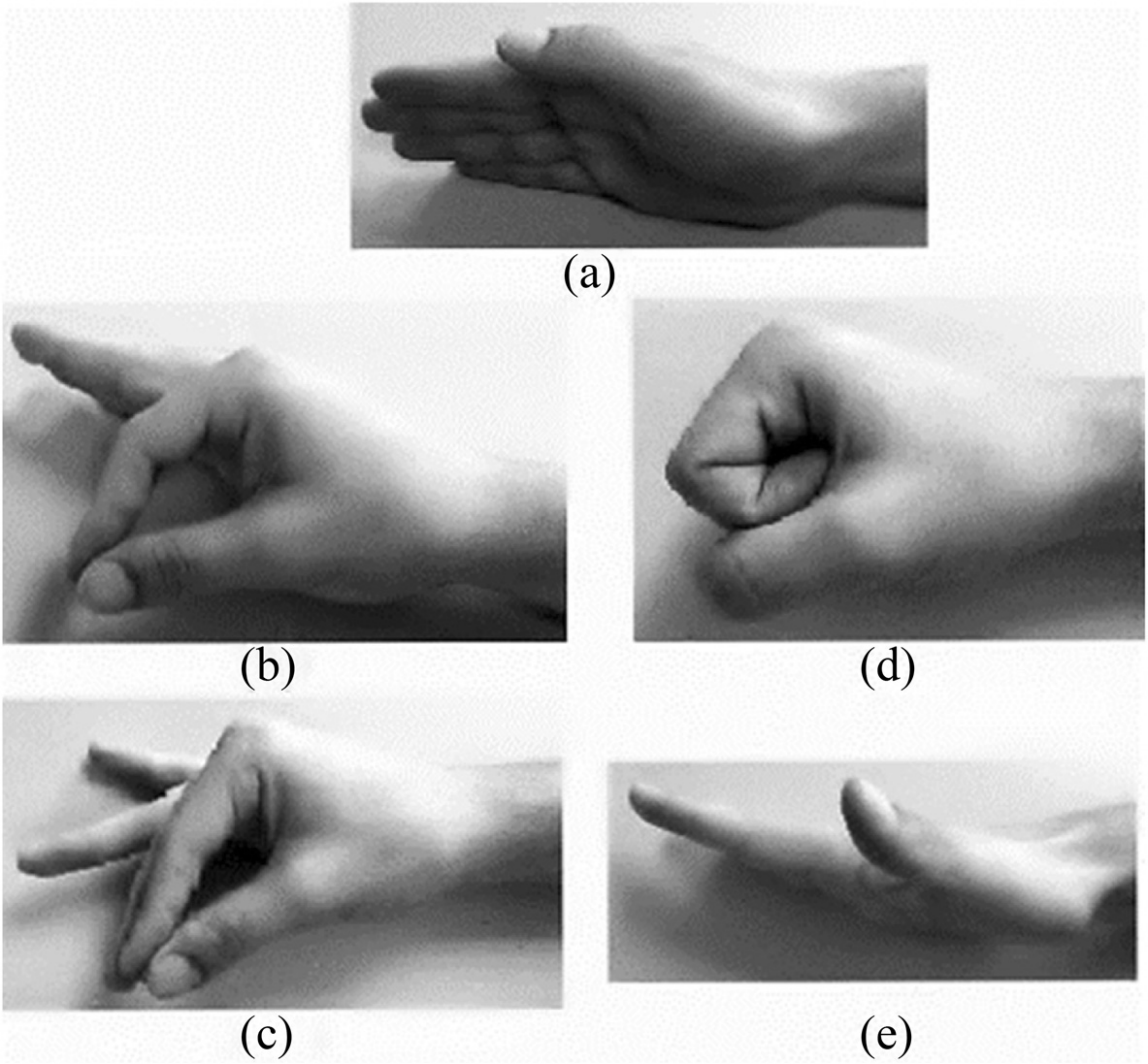
**Hand positioning: (a) Neutral position, (b) Pinch, (c) Tripod, (d) Hand close, (e) Hand extended.**

The participants were seated with their forearms rested and arms flexed with elbow at 90°. A total of ten gestures were considered, which can be broadly considered as five finger and five hand grip flexion gestures, and these are shown in Figures 2 and 3. The five functional grips were; (i) Neutral position, (ii) Pinch, (iii) Tripod pinch with index, middle finger and thumb, (iv) Hand close and (v) Hand open.

Verbal and visual cue were given to the participants for them to perform the different gestures. The duration of each gesture was more than 1 second, and the movements were repeated thirty times, with the order of the movements being random. Participants were requested to perform the gestures at their comfortable pace and to rest between each trial.

### Data analysis

Power Spectral Density Average (PSD-Av) was selected as the signal feature because this has been considered to be robust and suitable for easy implementation [22]. This was computed for each gesture with a window of 1 second and for all the five sEMG channels. This resulted in a vector with five PSD-Av values for each gesture example. Data analysis was performed for each subject individually and averaged for the four subjects. This was in line with the standard protocol used for myoelectric prosthetic control, where the system is custom trained for each user.

Leave one our cross-validation technique has been well accepted for stable algorithms and databases. The advantage of using leave one out cross validation method is that it is suitable for generalization of the results by using different sets of training and testing datasets. This validation method tests the training and testing relationship for each possible set combination and thus overcomes any possible bias in the classification [23]. Using leave one out approach, the thirty examples were divided in two sets; training and test datasets. The PSD-Av for the five channels was the input to a Fisher’s Linear Discriminant Classifier [24,25]. This was trained for each person separately and for the 10 classes. Using leave one out approach, this was repeated thirty times for each subject. Average accuracy, sensitivity and specificity were calculated and the confusion matrix was generated. The rows correspond to the priori, the columns are the predictions and the diagonal corresponds to the correct predictions [26,27].

The average accuracy is *A*_*CC*_, and is the proportion of correct prediction:

1 $$ {A}_{CC}=\frac{{\displaystyle {\sum}_{i=1}^g}{C}_{ii}}{{\displaystyle {\sum}_{i=1}^g}{\displaystyle {\sum}_{j=1}^g}{C}_{ij}} $$

where, *C*_*i j*_ corresponds to the Confusion Matrix element in row *i* and column *j* while *g* is the total number of classes.

Measurement of the error or accuracy is not sufficient to determine the suitable class because these do not consider the misclassifications, and there can be systematic error bias [26] and hence an approach that considered the false negatives and false positives was proposed. A new method has been developed that identifies the least suitable hand-gestures based on the ranking obtained from the confusion matrix using a combination of false-negatives and false positives.

The Sensitivity refers to the ability of the test to correctly identify the condition. Thus, in the current example:

2 $$ Se = \frac{True\  Positive}{True\  Positive+ False\  Negative} $$

The Specificity corresponds to the test ability to exclude a condition correctly. In the present example, it can be defined by:

3 $$ Sp=\frac{True\  Negatives}{True\  Negatives+ False\  Positives} $$

The gestures that are most suitable are the ones that have high authentic correct and least false identification. For this aim, the results were used to populate the confusion matrix. In this proposed technique, the confusion matrix was multiplied with a diagonal matrix where all the diagonal elements were unity, and the other elements were negative 1 (refer equation 4). This method inverts the sign of all the cells of the matrix expect the diagonal members.

The difference between the diagonal cells and the other cells of each row indicates the false negative prediction (FNP), while the difference between the diagonal element and the other cells in each column indicates the false positive predictions (FPP). These were used to obtain the Positive–Negative Performance Measurement Index (PNM). This considers the correct classification as the positive, misclassification caused due to its presence as negative, and the summation of these two indicates the PNM. The novelty of this measure is that unlike other studies that only consider the accuracy; this identifies the sensitivity and specificity of the system, thereby identifying those gestures that lead to overall improvement.

The PNM measures the combined misclassification in the prediction of each class (gesture). The first part of the index considers the FNP while the second the FPP. The index ranges from 1 if all predictions are corrects to −1 if all predictions are wrongs.

4 $$ PN{M}_i=\frac{\left({C}_{ii}-{\displaystyle {\sum}_{j\ne i}^g}{C}_{ij}\right) + \left({C}_{ii}-{\displaystyle {\sum}_{i\ne j}^g}{C}_{ij}\ \right)}{{\displaystyle {\sum}_{i,j=1}^g}{C}_{ij}+{\displaystyle {\sum}_{j,i=1}^g}{C}_{ij}} $$

where, *C*_*i j*_ corresponds to the Confusion Matrix element in row *i* and column *j* while *g* is the total number of classes.

The classes were ranked based on the PNM, and the one which has the smallest value was rejected. After removing the lowest ranked gesture, the data was analyzed again to obtain the new ranking and accuracies. This process was repeated till misclassification was less than 5%. This is an iterative method using the new proposed index called PNM.

## Results and discussion

The Confusion Matrix is a table where row elements correspond to the response of each class while the column is the number of times the class was predicted, while the diagonal matrix elements show the number of times the class was correctly identified. The last column lists the percentage accuracy for each class.

Table 1 shows the confusion matrix for the ten gestures, along with the number of FPP and FNP as defined before. Table 2 shows the PNM (equation 4) of all the classes where the classes have been ranked according these values.

**Table 1:** **Confusion Matrix for functional hand gesture and finger flexion recognition system**

**Classes**		**1**	**2**	**3**	**4**	**5**	**6**	**7**	**8**	**9**	**10**	**Acc**	**FNP**
**1**	**Neutral**	70	20	8	13	4	0	4	1	0	0	58	20
**2**	**Thumb**	16	97	0	0	0	0	3	3	1	0	81	74
**3**	**Index**	4	0	98	3	2	0	6	5	2	0	82	76
**4**	**Middle**	13	0	4	88	3	0	0	11	0	1	73	56
**5**	**Ring**	3	0	0	1	114	1	0	1	0	0	95	108
**6**	**Little**	4	2	0	2	4	105	1	2	0	0	88	90
**7**	**Pinch**	4	8	7	0	0	0	89	9	3	0	74	58
**8**	**Tripod**	1	1	4	9	0	4	9	86	6	0	72	52
**9**	**Hand Close**	0	0	9	0	2	0	5	8	96	0	80	72
**10**	**Hand Open**	0	0	0	0	0	0	1	2	0	117	98	114
	**FPP**	25	66	66	60	99	100	60	44	84	116		
	**Average Accuracy**											80.0	
	**Average Sensitivity**											80.0	
	**Average Specificity**											97.8

**Table 2:** **Positive–Negative Performance Measurement Index ranking each class based on the Confusion Matrix of the 10-classes**

**Classes**		**PNM**
1	Hand Open	0.97
2	Ring	0.83
3	Little	0.83
4	Hand Close	0.68
5	Index	0.57
6	Thumb	0.56
7	Pinch	0.50
8	Middle	0.49
9	Tripod	0.41
10	Neutral	0.19

From Table 1, it is observed that the average accuracy is 80.0%, making such a system unsuitable for most prosthetic applications. After ranking the gestures in term of the PNM (Table 2), the ‘Neutral’ gesture was removed and the analysis repeated. After removing this class, the average accuracy of balance 9-classes improved to 84.8% and the new ranking shown in Table 3.

**Table 3:** **Positive–Negative Performance Measurement Index ranking each class based on the Confusion Matrix of the 9-classes**

**Classes**		**PNM**
1	Hand Open	0.97
2	Ring	0.85
3	Thumb	0.83
4	Little	0.79
5	Middle	0.68
6	Hand Close	0.64
7	Index	0.61
8	Pinch	0.55
9	Tripod	0.36

In Table 3, the Tripod has the lowest rank. Removing this and repeating the analysis improved the accuracy to 89.1% and the new ranking is shown in Table 4. The lowest ranked gesture in this table was the pinch, and removing this improved the accuracy to 93.7%. The resultant confusion matrix is shown in Table 5, while the PNM values and the ranking of the gestures shown in Table 6. Repeating the process once again resulted in the list of best 6 gestures (Table 7); Hand open, Ring finger flexion, Hand closed, Thumb flexion, Little finger flexion, and Middle finger flexion. The average accuracy of the best six hand gestures is 96.5% (Table 8).

**Table 4:** **Positive–Negative Performance Measurement Index ranking each class based on the Confusion Matrix of the 8-classes**

**Classes**		**PNM**
1	Hand Open	0.95
2	Ring	0.88
3	Thumb	0.83
4	Middle	0.82
5	Hand Close	0.77
6	Little	0.75
7	Index	0.65
8	Pinch	0.60

**Table 5:** **Confusion Matrix for the most feasible 7-classes**

**Classes**		**1**	**2**	**3**	**4**	**5**	**6**	**7**	**Acc %**
**1**	**Thumb**	115	1	0	1	0	3	0	96
**2**	**Index**	2	110	3	0	1	4	0	92
**3**	**Middle**	1	6	110	1	2	0	0	92
**4**	**Ring**	0	0	2	117	1	0	0	98
**5**	**Little**	0	1	4	2	110	3	0	92
**6**	**Hand Close**	0	9	1	2	1	107	0	89
**7**	**Hand Open**	1	0	0	0	0	1	118	98
	**Average Accuracy**								93.7
	**Average Sensitivity**								93.7
	**Average Specificity**								96.9

**Table 6:** **Positive–Negative Performance Measurement Index ranking each class based on the Confusion Matrix of the 7-classes**

**Classes**		**PNM**
1	Hand Open	0.98
2	Ring	0.93
3	Thumb	0.92
4	Little	0.87
5	Middle	0.83
6	Hand Close	0.80
7	Index	0.78

**Table 7:** **Positive–Negative Performance Measurement Index ranking each class based on the Confusion Matrix of the 6-classes**

**Classes**		**PNM**
1	Hand Open	0.97
2	Ring	0.95
3	Hand Close	0.93
4	Thumb	0.93
5	Little	0.91
6	Middle	0.88

**Table 8:** **Confusion Matrix for the most feasible 6-classes**

**Classes**		**1**	**2**	**3**	**4**	**5**	**6**	**Acc %**
**1**	**Thumb**	116	1	0	0	3	0	97
**2**	**Middle**	1	115	1	2	1	0	96
**3**	**Ring**	0	2	118	0	0	0	98
**4**	**Little**	1	5	2	112	0	0	93
**5**	**Hand Close**	1	1	1	0	117	0	98
**6**	**Hand Open**	2	1	0	0	1	116	97
	**Average Accuracy**							96.5
	**Average Sensitivity**							96.5
	**Average Specificity**							99.3

To obtain high sensitivity and specificity comes at the cost of reducing the number of gestures, reduced from 10 to 6. While this may be restrictive in some circumstances, however, this significantly lowers the misclassifications and improves the accuracy leading to greater reliability and safety for the user. High misclassification for some of the gestures may be attributed to the complexity of the hand anatomy and the required co-activation of number of muscles for hand grip and finger flexion.

The outcome of these experiments may be dependent on the initial gesture set and the gesture recognition system such as number of channels, the signal features and also the classifier. However, this work has shown that there is the need for appropriate selection of gestures for reliable prosthetic control, and this selection may be achieved using PNM. The significance of this method is that it has introduced a new performance index for identifying the most suitable gestures for myoelectric prosthetic hand control. This index is based on measuring both, the total number correct classification and the misclassifications.

This work has used sEMG analysis and classification methods reported in literature and hence no effort has been made to validate their efficacy because this has been reported in literature. There may be differences in the gestures that may be selected if the signal analysis and classification method would be different, however, this work has shown that the selection of appropriate gestures is extremely important, and the proposed method is effective for this purpose. While there may be differences between the desired gestures between applications, this work has highlighted the need for selecting the gestures based on the required system sensitivity and specificity.

## Conclusion

The work has found that when a set of 10 hand gestures; five individual finger flexion and five functional hand grips, were considered, the accuracy, sensitivity and specificity were 80.0%, 80.0% and 97.8% respectively (Table 1). With the same set of signal features and classification techniques, and an appropriate selection of gestures using PNM iterative method, the accuracy, sensitivity and specificity improved to 96.5%, 96.5% and 99.3%.

The novelty of this work is the development of a method to select the most suitable hand gestures that improve the sensitivity and specificity of the myoelectric hand gesture recognition system for safer prosthetic hand control. The work has used well established sEMG analysis and classification techniques, which was chosen as an example, and based on favorable literature review, and the authors do not take any credit for this.

This work has shown that it is important to select the set of hand-gestures that will be accurately recognized for a reliable myoelectric based prosthetic hand or other human computer interface systems. While the selection of the gestures is based on the specific application and may be different for different sEMG analysis and classification techniques, the outcome of this work shows that the selection should be performed based on the ranking of the sensitivity and specificity. This would identify those gestures that would lead to high error and thus should be discarded. In this study from an initial set of ten hand gestures, which included five finger flexion and five functional hand gestures, a set of six gestures were identified which gave the sensitivity and specificity greater than 95%.

This work has demonstrated the principle for selecting appropriate gestures based on the accuracy, sensitivity and specificity of myoelectric based hand gesture recognition. While the hypothesis has been tested using Power Spectral Density Average (PSD-Av) and linear classifier, for generalizing this principle, this hypothesis has to be tested for other features and classifiers. This may result in different number of gestures that provide the suitable sensitivity and specificity.

## Abbreviations

sEMG: Surface electromyography
PSD: Power spectral density
PSD-Av: Power spectral density average
PNM: Positive – Negative Performance Measure Index
FNP: False negative predictions
FPP: False positive predictions
SENIAM: Surface ElectroMyoGraphy for the non-invasive assessment of muscle
